# Supplementary material for: Probabilistic adaptation in changing microbial environments
Source: PeerJ. 2016 Dec 14;4:e2716. doi: 10.7717/peerj.2716 (PMC5160922; doi:10.7717/peerj.2716)
Supplement: Algorithm S1 [file peerj-04-2716-s001.pdf]

Algorithm S1 Particle filtering algorithm for real-time inference in meta-changing environments.

```
initialize N particles P := p_1, ..., p_N from prior
each p_i = {s_i, S_i, C_i}, where s_i is switch state value, S_i switch transition matrix, C_i nutrient transition array
initialize particle weights W := w_1, ..., w_N uniformly, w_i := 1/N
while next nutrient C_t do
    for each particle p_i do
        weigh particle by likelihood of observed nutrient, w_i := P(C_t | C_{t-1}, S_t = s_i, S_i, C_i)
        update nutrient transition array C_i
    resample particles by weights, P := RESAMPLE(P, W)
    reset weights, w_i := 1/N
    for each particle p_i do
        sample new particle state for t + 1, p_i := P(S_{t+1} | S_t, S_i)
        update particle switch transition matrix S_i
```
