# Supplementary material for: Probabilistic adaptation in changing microbial environments
Source: PeerJ. 2016 Dec 14;4:e2716. doi: 10.7717/peerj.2716 (PMC5160922; doi:10.7717/peerj.2716)
Supplement: Table S1 — Equations with all rate parameters are available in 10.6084/m9.figshare.3492185. [file peerj-04-2716-s002.pdf]

1	$\mathsf{Glu} \longrightarrow \varnothing$
2	$\mathsf{Gal} \longrightarrow \varnothing$
3	$\mathsf{Glu\_Sensor} \longrightarrow \varnothing$
4	$\mathsf{Gal} \longrightarrow \mathsf{Glu\_Sensor} + \mathsf{Gal}$
5	$\mathsf{Gal\_Sensor} \longrightarrow \varnothing$
6	$\mathsf{Gal\_Sensor} + \mathsf{Gal} \rightleftharpoons \mathsf{Gal\_Activator}$
7	$\mathsf{Glu} \longrightarrow \mathsf{Gal\_Sensor} + \mathsf{Glu}$
8	$\mathsf{Gal\_Activator} \longrightarrow \mathsf{Glu\_To\_Gal} + \mathsf{Gal\_Activator}$
9	$\mathsf{Glu\_To\_Gal} \longrightarrow \varnothing$
10	$\mathsf{Gal\_To\_Glu} \longrightarrow \varnothing$
11	$\mathsf{Glu\_Activator} \longrightarrow \mathsf{Gal\_To\_Glu} + \mathsf{Glu\_Activator}$
12	$\mathsf{Glu\_Sensor} + \mathsf{Glu} \rightleftharpoons \mathsf{Glu\_Activator}$
