# Supplementary figures and images for: Probabilistic adaptation in changing microbial environments
Source: PeerJ. 2016 Dec 14;4:e2716. doi: 10.7717/peerj.2716 (PMC5160922; doi:10.7717/peerj.2716)

**A**

Growth rates on different sugars (61 yeast strains)

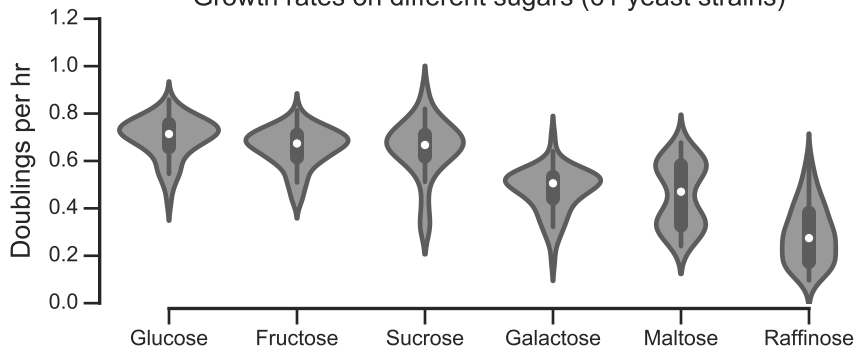**B**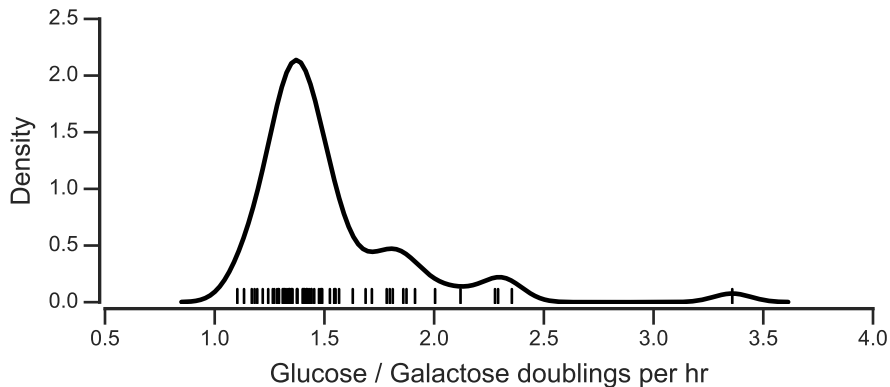

Supplement: Figure S1 — (A) Mean growth rates (doublings per hour) from two replicate cultures grown with different sugars as primary carbon source. (B) Distribution of the ratio of glucose to galactose growth rate for 61 yeast strains. [file peerj-04-2716-s003.pdf]

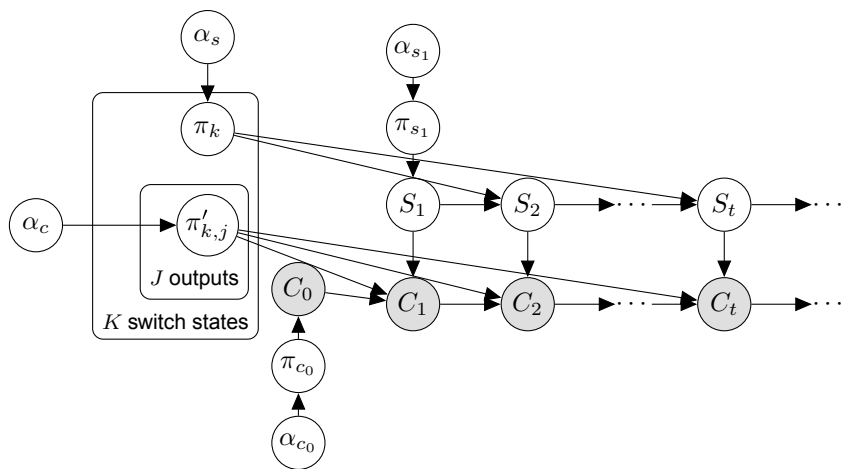

Supplement: Figure S2 — All random variables and hyperparameters shown. Model drawn using plate notation. (This model is similar to an Autoregressive HMM.) [file peerj-04-2716-s004.pdf]

**A**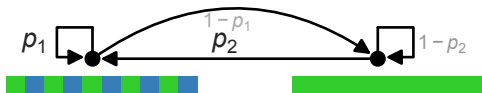**B**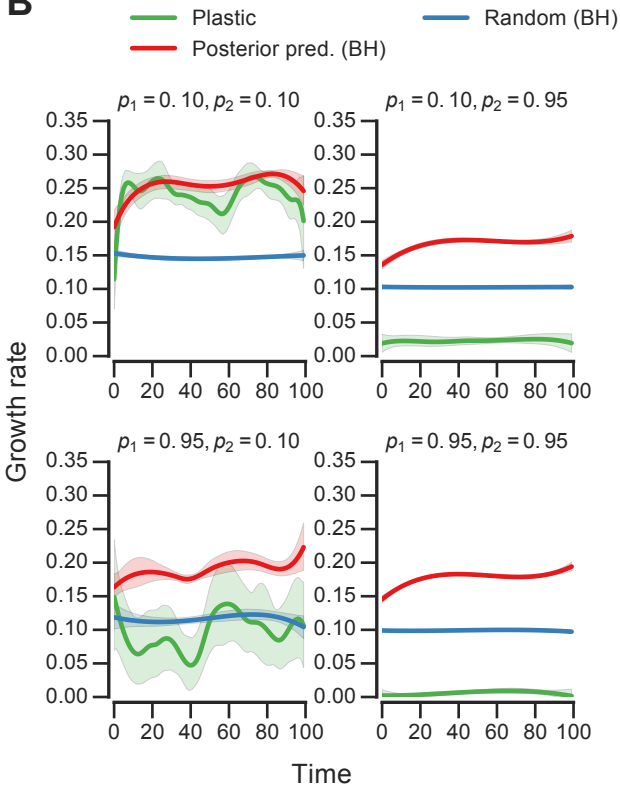

Supplement: Figure S3 — (A) Meta-changing environment (same as Figure 5A). (B) Growth rates obtained using different growth policies in meta-changing environment shown in (A). ”Posterior pred. (BH)” indicates a bet-hedging policy where fraction of population tuned to a nutrient is set by the real-time estimate of the posterior predictive probability of the nutrient, ”Random (BH)” indicates a bet-hedging policy where fraction of population tuned to nutrient is set randomly. ”Plastic” policy is a non-bet-hedging policy plotted for reference (same as Figure 5B). Mean growth rates from 20 simulations plotted with bootstrap confidence intervals (shaded regions). [file peerj-04-2716-s005.pdf]
